# Stent interventions for pulmonary artery stenosis improve bi-ventricular flow efficiency in a swine model

**DOI:** 10.1186/s12968-021-00709-4

**Published:** 2021-02-25

**Authors:** Ryan J. Pewowaruk, Gregory P. Barton, Cody Johnson, J. Carter Ralphe, Christopher J. Francois, Luke Lamers, Alejandro Roldán-Alzate

**Affiliations:** 1 Biomedical Engineering, University of Wisconsin-Madison, Madison, WI USA; 2 University of Wisconsin-Madison, Madison, WI USA; 3 Medical Physics, University of Wisconsin-Madison, Madison, WI USA; 4 School of Medicine and Public Health, University of Wisconsin-Madison, Madison, WI USA; 5 Division of Cardiology, University of Wisconsin-Madison, Madison, WI USA; 6 Mechanical Engineering, University of Wisconsin-Madison, Madison, WI USA

**Keywords:** 4D flow MRI, Congenital heart disease, Pediatrics, Right ventricle

## Abstract

**Background:**

Branch pulmonary artery (PA) stenosis (PAS) commonly occurs in patients with congenital heart disease (CHD). Prior studies have documented technical success and clinical outcomes of PA stent interventions for PAS but the impact of PA stent interventions on ventricular function is unknown. The objective of this study was to utilize 4D flow cardiovascular magnetic resonance (CMR) to better understand the impact of PAS and PA stenting on ventricular contraction and ventricular flow in a swine model of unilateral branch PA stenosis.

**Methods:**

18 swine (4 sham, 4 untreated left PAS, 10 PAS stent intervention) underwent right heart catheterization and CMR at 20 weeks age (55 kg). CMR included ventricular strain analysis and 4D flow CMR.

**Results:**

4D flow CMR measured inefficient right ventricular (RV) and left ventricular (LV) flow patterns in the PAS group (RV non-dimensional (n.d.) vorticity: sham 82 ± 47, PAS 120 ± 47; LV n.d. vorticity: sham 57 ± 5, PAS 78 ± 15 *p* < 0.01) despite the PAS group having normal heart rate, ejection fraction and end-diastolic volume. The intervention group demonstrated increased ejection fraction that resulted in more efficient ventricular flow compared to untreated PAS (RV n.d. vorticity: 59 ± 12 *p* < 0.01; LV n.d. vorticity: 41 ± 7 *p* < 0.001).

**Conclusion:**

These results describe previously unknown consequences of PAS on ventricular function in an animal model of unilateral PA stenosis and show that PA stent interventions improve ventricular flow efficiency. This study also highlights the sensitivity of 4D flow CMR biomarkers to detect earlier ventricular dysfunction assisting in identification of patients who may benefit from PAS interventions.

## Introduction

Branch pulmonary artery (PA) stenosis (PAS) commonly occurs in patients with complex congenital heart disease (CHD). Acute post-surgical PAS is associated with hemodynamic instability, prolonged in-hospital recovery and increased mortality [1–5]. Chronic PAS is associated with abnormal PA growth and pulmonary blood flow mal-distribution and can lead to PA hypertension [6, 7]. These vascular pathologies contribute to reduced exercise capacity and progressive right ventricular (RV) dysfunction thought to be related to chronic ventilation-perfusion mismatch and increased RV afterload [8]. For many patients the first line therapy for treatment of PAS includes catheter interventions with balloon angioplasty and intravascular stenting. Interventional outcome measures have long focused on the technical and anatomic success of the procedure, defined principally as an immediate post-procedure increase in PA size [9]. Thus far, there has been limited information detailing the impact of intravascular stenting on the ventricular response to these interventions.

In CHD patients with PAS standard measures of ventricular function are often normal until late in the disease process even with significant pulmonary blood flow mal-distribution [9] and indications for PAS interventions are not clearly defined. Ventricular flow analysis using four dimensional (4D) flow cardiovascular magnetic resonance (CMR; 4D flow CMR) is an increasingly utilized method to study ventricular function in patients with CHD before and after complex interventions [10–14]. Measurement of time-varying velocity of blood flow with 4D flow CMR can precisely define ventricular flow patterns and permit calculation of biomarkers of mechanical efficiency including kinetic energy and vorticity [15, 16]. Changes in these ventricular flow biomarkers are believed to represent early signs of cardiac dysfunction [17] as momentum lost through inefficient ventricular flow increases ventricular energy demand and contributes to adverse ventricular remodeling [18, 19]. We believe the advanced imaging capabilities of 4D flow CMR can detect early markers of ventricular dysfunction caused by PAS and may assist in identifying patients who could benefit from PAS interventions.

With evidence that 4D flow CMR detects early biomarkers of ventricular dysfunction, we performed this study applying CMR ventricular flow analysis in a swine model of treated and untreated unilateral branch pulmonary artery stenosis and compared the 4D flow data to conventional anatomic and hemodynamics measures. We hypothesized that untreated PAS is associated with abnormal and inefficient RV flow patterns that are quantifiable by 4D flow CMR and that PAS interventions with intravascular stenting lead to improvement in RV mechanics.

## Methods

### Experimental protocol

Isolated left PAS was created in neonatal piglets (n = 14, 5. 4 kg, 2 weeks of age) by suturing a short segment of 4.0 mm Gore-Tex tube graft around the proximal left PA (LPA) [20] with 4 additional piglets having sham surgery to serve as normal controls. Four swine served as untreated left PAS controls and 10 swine underwent stent interventions to treat the left PAS. Stent interventions occurred between 5 and 10 weeks of age with a possible redilation procedure to match the distal LPA diameter by 10 weeks of age. Further technical details for this animal model of left PAS and stent interventions are detailed in our previous publication [20]. All groups (sham: n = 4, left PAS n = 4, and intervention n = 10) had right heart catheterizations (RHC), computed tomography (CT) angiography and CMR at 20 weeks of age. Given the faster rate of cardiopulmonary development in swine compared to humans [21], this timeline is developmentally equivalent to performing stent interventions in a 3–5 year old human patient and then comparing outcomes in late adolescence or early adulthood. This study complied with all institutional and national requirements for the care and use of laboratory animals and the Institutional Animal Care and Use Committee of the University of Wisconsin reviewed and approved this protocol.

### Hemodynamic measurements at 20-week catheterization

RHC was performed following induction (telazol and xylazine intramuscular) and anesthesia (isoflurane 2–5%) during mechanical ventilation**.** Venous access was obtained percutaneously and intravenous heparin was given. Beat-by-beat pressure waveforms were obtained in the right atrium, RV, main PA, right PA (RPA), LPA, and bilateral capillary wedge positions using end-hole fluid filled catheters attached to a pressure transducer.

### Computed tomography angiography

**Fig. 1:**
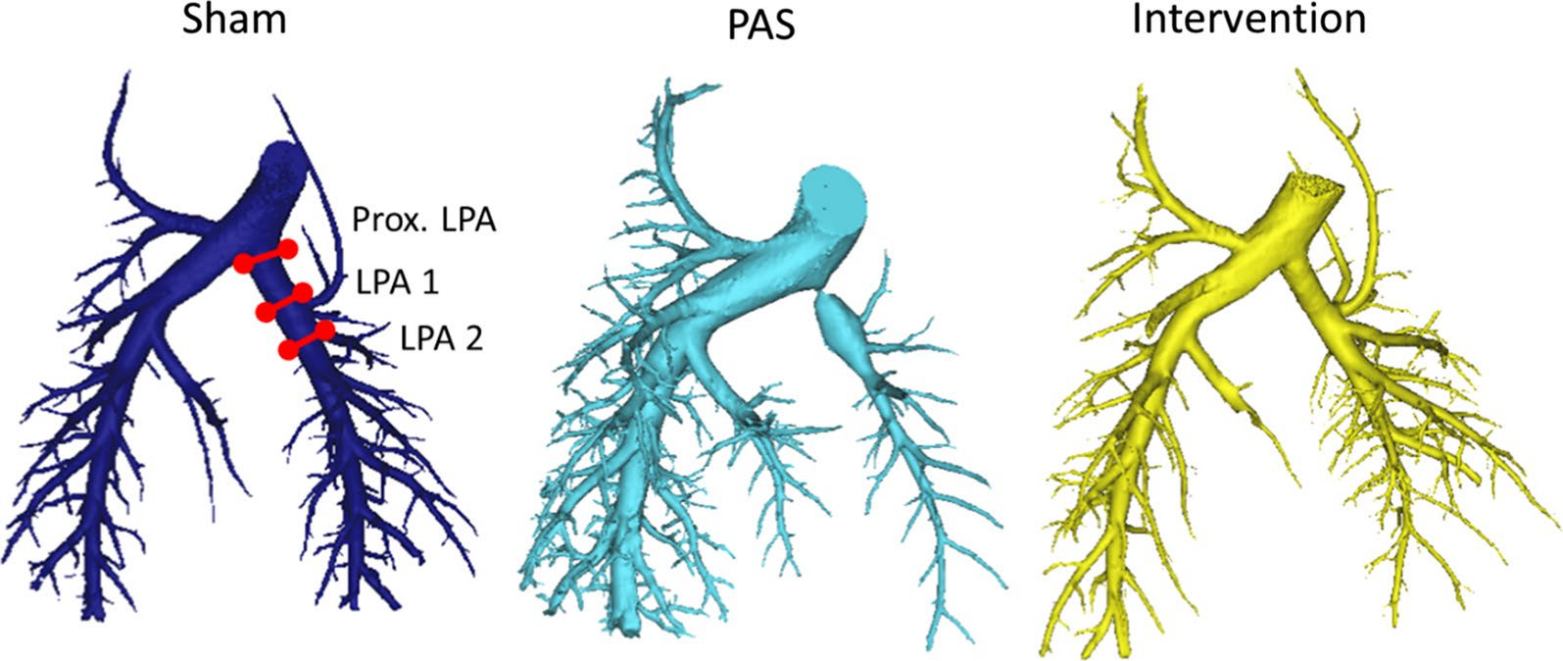
Representative 3D pulmonary artery reconstructions for sham, pulmonary artery (PA) stenosis (PAS) and stent interventions. The left pulmonary artery (LPA) diameter measurement locations are shown on the sham PAs

Under the same anesthesia, CT angiography was performed using a 64-slice CT scanner (750 CT, General Electric Healthcare, Waukesha, Wisconsin, USA) with retrospective cardiac gating and intravenous Iopamidol-370 (Bracco Diagnostics Inc., Monroe Township, New Jersey, USA) for contrast enhancement. The LPA diameter was measured immediately after the PA bifurcation and at two more distal locations adjacent to the LPA first order branches as previously defined [20]. (See Fig. 1 for measurement locations and representative 3D PA reconstructions).

### CMR protocol

Under the same anesthesia on a 3T system (Discovery MR 750, General Electric Healthcare) using a short axis balanced steady state free precession (bSSFP) CMR sequence and the under sampled-radial 4D flow CMR sequence PC-VIPR (Phase Contrast Vastly Under sampled Projection Imaging) [22, 23]. bSSFP CMR used a 50° flip angle, TR = 3.3 ms, TE = 1.3 ms, a voxel size of 1.4 × 1.4x8.0 mm and data was reconstructed to 20 time frames per cardiac cycle. 4D flow CMR acquisition parameters include TR = 6.3 ms, TE = 2.1 ms, an isotropic voxel size of 1.25 × 1.25x1.25 mm, VENC = 150 cm/sec and data was reconstructed to 20 time frames per cardiac cycle. 4D flow CMR was used to evaluate ventricular flow.

### Cine CMR analysis

Ventricular volumes were segmented and ejection fraction (EF) and cardiac output (CO) were calculated and normalized to body surface area (BSA). Interventricular septum (IVS) curvature was calculated from the IVS radius at a mid-ventricular slice normalized by the cube root of LV end-diastolic volume (EDV).

Strain was calculated from short axis CMR using feature tracking in Segment 2.2 R6423 (http://segment.heiberg.se/, [24]). Global peak circumferential systolic strain, peak circumferential systolic strain rate and peak circumferential diastolic strain rate were calculated for both the left ventricle (LV) and the RV. One stenosis swine was excluded from strain analysis because of poor image quality.

### 4D flow CMR analysis

Lung perfusion was quantified from 4D flow CMR using flow visualization software (EnSight, Ansys, Canonsburg, Pennsylvania, USA). LPA flow rate was calculated by subtracting the RPA flow rate from the main PA flow rate as CMR signal is lost in a metal stent and distal to the stenosis there may be aliasing dephasing. A 4D flow CMR dataset was not obtained for one intervention swine.

The LV and RV were segmented from end diastolic cine frames. The end diastolic frame was chosen to capture the ventricles through the entire cardiac cycle. The use of a single ventricle mask has previously been validated against time resolved masks with a single ventricle mask mildly overestimating peak systolic kinetic energy [25]. Velocity field gradients were calculated using a centered finite difference in MATLAB (Mathworks, Natick, Massachusetts, USA). The density and viscosity of blood were assumed to be 1.06 g/cm^3^ and 0.04 poise respectively.

Using the velocity data from the 4D flow CMR acquisitions, kinetic energy (KE), vorticity (${\varvec{\omega}}$) and energy dissipation rate ($\varepsilon$) were calculated from the following equations

1 $$KE = \frac{1}{2}\rho {\varvec{u}}^{2}$$

2 $${\varvec{\omega}} = \nabla \times {\varvec{u}}$$

3 $$\varepsilon = 2\mu \left( {\varvec{S}} \right)^{2}$$

where $\rho$ is blood density, ***u ***is the velocity vector, $\nabla \times$ is = the curl operator, $\mu$ is blood viscosity and ***S*** is the strain rate tensor (${\varvec{S}}=\frac{1}{2}\left[\nabla {\varvec{u}}+\nabla {{\varvec{u}}}^{T}\right]$). Flow parameters were reported similar to prior studies [26, 27]: KE was reported at peak systole and peak diastole, vorticity was averaged over the cardiac cycle and energy dissipation rate was summed over the cardiac cycle to calculate the total energy dissipation. Both KE and energy dissipation were normalized by stroke volume.

Ventricular flow parameters are typically either not normalized or normalized by stroke volume although heart rate and ventricular volume could also be different be subjects. To account for these differences, dimensionless KE, vorticity magnitude and energy dissipation rate were calculated from dimensional analysis with characteristic length and velocity scales calculated from CO and EDV.

4 $$KE^{*} = \frac{KE}{{\rho CO^{2} EDV^{{ - \frac{4}{3}}} }} = \frac{KE}{{\rho \left( {HR*SV} \right)^{2} EDV^{{ - \frac{4}{3}}} }}$$

5 $${\varvec{\omega}}^{*} = \frac{{\varvec{\omega}}}{{CO*EDV^{ - 1} }} = \frac{{\varvec{\omega}}}{{HR*SV*EDV^{ - 1} }}$$

6 $$\varepsilon^{*} = \frac{\varepsilon }{{\mu CO^{2} EDV^{ - 2} }} = \frac{\varepsilon }{{\mu \left( {HR*SV} \right)^{2} EDV^{ - 2} }}$$

where * refers to the dimensionless variable. Dimensional analysis is the proper fluid dynamics method to account for this variation in geometry and flow conditions.

Lastly, to understand the mechanism of ventricular energy dissipation we calculated the percentage of energy dissipation due to vorticity ($100\%* {{\varvec{\omega}}}^{2}/2{{\varvec{S}}}^{2}$) over the entire cardiac cycle to determine if energy dissipation was primarily due to vorticity or due to surface strain rate. As this is a novel biomarker for ventricular flow we additionally performed a “sanity check” by comparing 4D flow CMR measurements to the vorticity-energy dissipation rate relationship for an idealized-theoretical vortex ring. Theoretical vortex ring results were presented for a Reynold’s number of 900 which is both reasonable for ventricular flow [28] and the theoretical formulation has been experimentally validated for this Reynold’s number [29].

### Statistics

All results were reported as mean ± standard deviation (SD). Statistical analysis was performed SPSS (Statistical Package for the Social Sciences, International Business Machines, Inc., Armonk, New York, USA). Data normality was assessed with a Shapiro-Wilkes test. In normally distributed data, statistical comparisons were made by ANOVA with Tukey’s honestly significant difference procedure for post hoc comparisons. When data were not normally distributed, comparisons between groups were made using the non-parametric Kruskal–Wallis test with a Bonferroni correction for post-hoc comparisons. The p-values for every comparison performed can be found in Appendix 1.

## Results

### Cardiac catheterization

Conventional hemodynamic measurements are summarized in Table 1. At 20 weeks, there were no differences in body weight, heart rate or RV/(LV + Septum) ratio. LPA banding created severe angiographic left PAS associated with a peak systolic main PA to distal LPA pressure gradient of 23 + 4 mmHg. The left PAS group had elevated mean right atrial, RV systolic, mean main PA pressure, main PA and right PA systolic pressures. The left PAS group also had decreased LPA systolic pressure.

**Table 1:** Hemodynamic measurements

	Sham	Left PAS	Intervention
Body weight (kg)	56 ± 7	57 ± 5	53 ± 10
Heart rate (bpm)	88 ± 8	84 ± 6	94 ± 15
RV/(LV + Septum) (g/g)	0.42 ± 0.03	0.40 ± 0.04	0.43 ± 0.04
Right atrial pressure (mmHg)	7 ± 2	10 ± 1	6 ± 1^#^
RV Systolic pressure (mmHg)	28 ± 4	38 ± 4*	28 ± 3^#^
Mean main PA pressure (mmHg)	19 ± 1	24 ± 1*	17 ± 1^#^
Main PA pressure (sys/dia, mmHg)	29 ± 3/14 ± 3	38 ± 4*/17 ± 2	26 ± 3^#^/13 ± 2
Right PA pressure (sys/dia, mmHg)	27 ± 3/15 ± 3	37 ± 3/18 ± 3	25 ± 3^#^/13 ± 3
Distal LPA pressure (sys/dia, mmHg)	28 ± 2/17 ± 2	15* ± 6/13 ± 5	23 ± 4^#^/14 ± 3
Proximal LPA pressure gradient (mmHg)	1 ± 2	23* ± 4	4 ± 4^#^
PCWP (mmHg)	10 ± 1	11 ± 3	8 ± 1^#^

*p < 0.05 vs. sham control, ^#^*p *< 0.05 vs. LPAS control, *sys* systolic, *dia* diastolic, *LPA* left pulmonary artery, *LV* left ventricle, *PA* pulmonary artery, *PCWP* pulmonary capillary wedge pressure, *RV* right ventricle

### Standard CMR and CT angiography

Results from standard CMR and CT angiography are shown in Table 2. Stent interventions significantly increased the size of the LPA at all measurement locations compared to the left PAS group (*p* < 0.01). Stent interventions did not increase the size of the proximal LPA (stented segment) or the first LPA measurement location (adjacent to the stent) to that of sham controls (*p* = 0.03 and *p* = 0.052). LPA diameters at the second, more distal, measurement location were similar between sham and stent interventions. Similarly, stenting increased left lung perfusion for the intervention group (42 ± 6%) compared to the left PAS group sixfold (7 ± 5%, *p* < 0.001) but did not reach perfusion levels of the sham control group (52 ± 9%, *p *= 0.07).

**Table 2:** Standard CMR and CT angiography

	Sham	Left PAS	Intervention
Proximal LPA:Aortic diameter (mm/mm)	1.26 ± 0.22	0.12 ± 0.01*	0.94 ± 0.21*^#^
LPA 1: Ao diameter (mm/mm)	1.19 ± 0.20	0.58 ± 0.23*	0.95 ± 0.11^#^
LPA 2: Ao diameter (mm/mm)	0.99 ± 0.10	0.52 ± 0.26*	0.94 ± 0.17^#^
L Lung Perfusion (%)	52 ± 9	7 ± 5*	42 ± 6^#^
CI (L/min/m^2^)	2.8 ± 0.5	3.2 ± 0.2	3.7 ± 0.8
RV SV/BSA (mL/m^2^)	33 ± 5	36 ± 5	36 ± 9
RV ESV/BSA (mL/m^2^)	50 ± 13	62 ± 16	35 ± 10^#^
RV EDV/BSA (mL/m^2^)	83 ± 13	98 ± 20	72 ± 15^#^
RV EF (%)	40 ± 7	37 ± 6	51 ± 8^#^
LV SV/BSA (mL/m^2^)	35 ± 8	41 ± 5	38 ± 10
LV ESV/BSA (mL/m^2^)	58 ± 4	62 ± 13	37 ± 10*^#^
LV EDV/BSA (mL/m^2^)	93 ± 9	102 ± 14	75 ± 12^#^
LV EF (%)	38 ± 6	40 ± 6	50 ± 10

*p < 0.05 versus sham control, ^#^*p* < 0.05 vs. *CI* cardiac index, *EDV* end-diastolic volume, *EF* ejection fraction,  *ESV* end-systolic volume, LPAS control, *SV* stroke volume, *BSA* body surface area

RV and LV stroke volume (SV) were not different between groups. The intervention group had smaller end systolic volumes (ESV) and EDV in both the RV and the LV compared to left PAS. The intervention group also had elevated RVEF versus left PAS (*p* = 0.03), trended towards increased RVEF versus sham (*p* = 0.06) and trended towards increased LVEF (*p* = 0.06) and increased cardiac index (CI) compared to the sham control group (*p* = 0.08).

### Ventricular strain analysis

Ventricular strain measurements did not identify any statistically significant differences between groups (Table 3).

**Table 3:** Ventricular strain analysis

	Sham	LPAS	Intervention
LV peak strain (%)	− 14.6 ± 2.7	− 15.6 ± 2.5	− 16.5 ± 2.9
LV peak diastolic strain rate (%/s)	85 ± 21	88 ± 24	109 ± 20
LV peak systolic strain rate (%/s)	− 74 ± 17	− 76 ± 18	− 90 ± 10
RV peak strain (%)	− 11.8 ± 4.5	− 12.5 ± 4.5	− 14.4 ± 5.6
RV peak diastolic strain rate (%/s)	46 ± 26	59 ± 21	59 ± 33
RV peak systolic strain rate (%/s)	− 59 ± 31	− 68 ± 30	− 82 ± 24

### Dimensional ventricular flow analysis

**Fig. 2:**
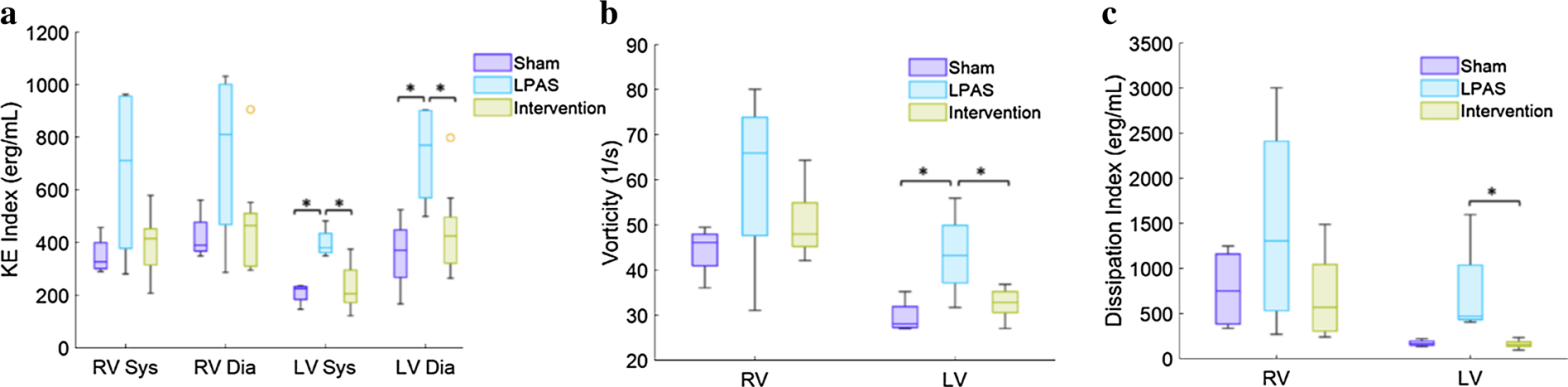
Dimensional RV and LV flow parameters. **a** peak systolic and peak diastolic kinetic energy (KE), **b** vorticity and **c** energy dissipation. Both KE and energy dissipation were indexed by stroke volume. *p < 0.05

KE, vorticity and energy dissipation indices are shown in Fig. 2. Stent interventions normalized all dimensional measures of ventricular mechanics to levels equal to sham controls. In the RV, the left PAS group trended towards increased systolic and diastolic KE, vorticity and energy dissipation although only systolic KE approached significance (p = 0.07 vs sham, p = 0.06 vs intervention). In the LV the left PAS group had increased systolic and diastolic KE, vorticity and energy dissipation.

### Dimensionless ventricular flow analysis

**Fig. 3:**
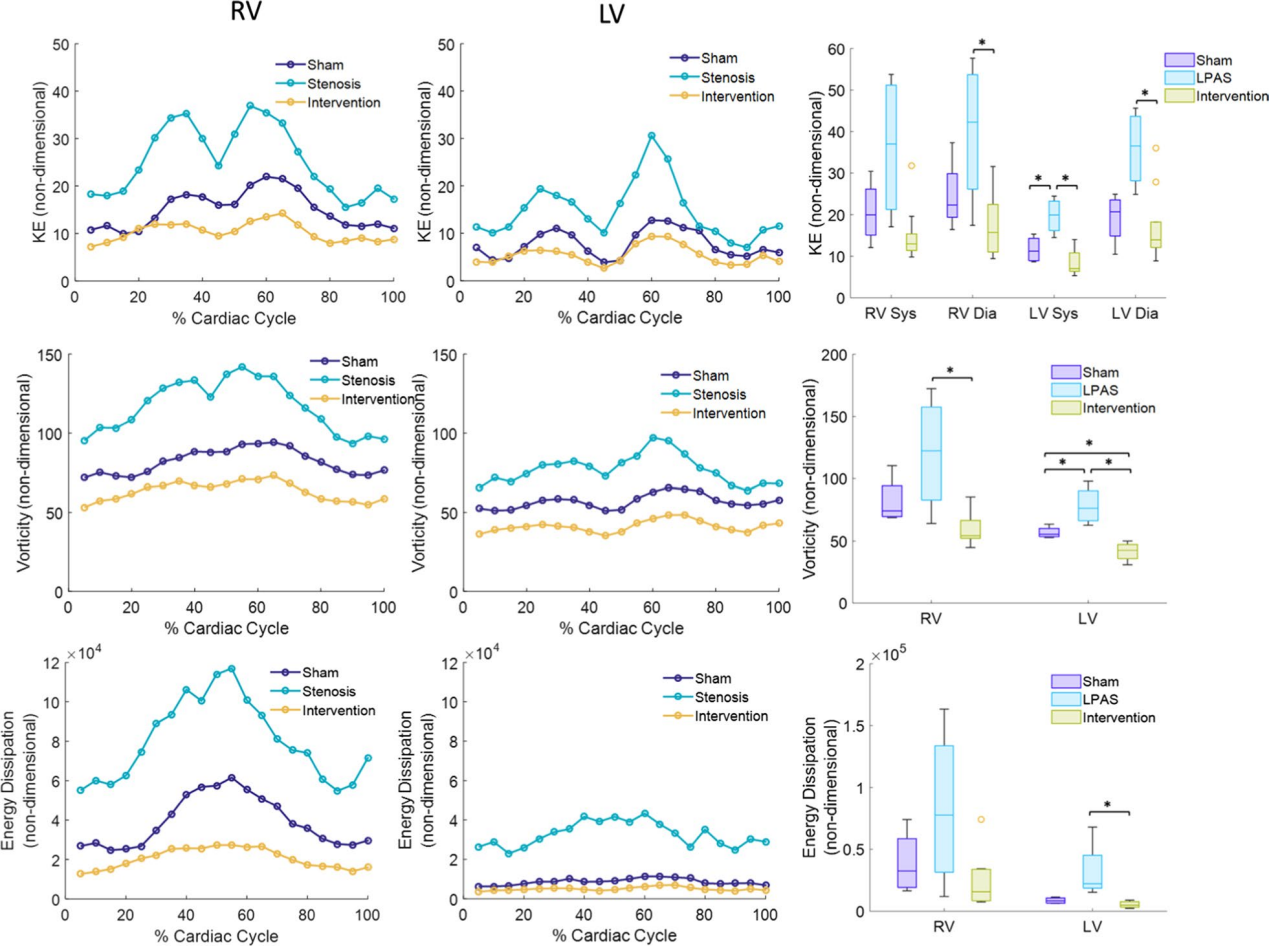
Time curves and bar graphs of non-dimensional flow parameters are shown for both the RV and the LV. Standard error is not shown on the time curve plots. Row 1: kinetic energy, Row 2: vorticity, Row 3: energy dissipation rate. Sys – systolic, Dia – diastolic. *p < 0.05

Time curves of dimensionless KE, vorticity and energy dissipation are shown along with bar graphs of systolic and diastolic peak KE, average vorticity and average energy dissipation rate in Fig. 3. The time curves demonstrate that throughout the cardiac cycle, the left PAS group has significant abnormalities in all measures of ventricular mechanics in both ventricles and stent interventions normalized all levels to that of sham controls. The analysis of dimensionless flow parameters again confirms the benefits of a PA stent intervention for improving the efficiency of ventricular flow. Elevated RV systolic KE, diastolic KE, and elevated RV vorticity in the left PAS group were not statistically significant when analyzing dimensional parameters, but were statistically significant when comparing groups using dimensionless parameters.

### Mechanism of energy dissipation

**Fig. 4:**
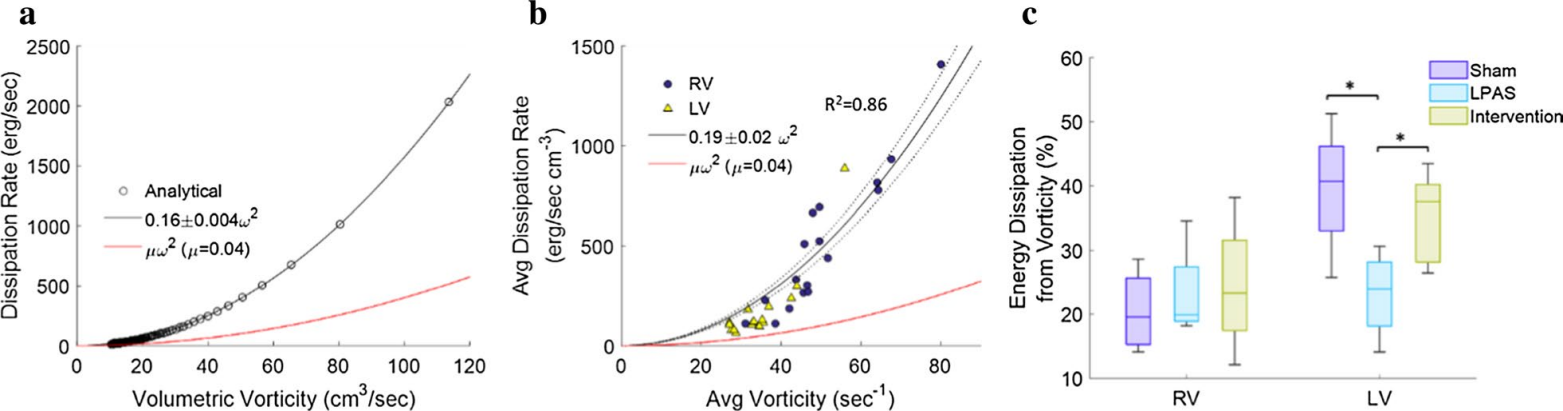
As expected, quadratic relationships are found between vorticity and energy dissipation rate for both (**a**). the idealized-theoretical vortex ring and (**b**). 4D flow CMR measurements of ventricular flow. **c** Percent energy dissipation from vorticity is compared between groups. *p < 0.05

For an idealized vortex ring [29], vorticity and energy dissipation rate are quadratically related with 25% of energy dissipation being directly due to vorticity (Fig. 4a). For all groups and both ventricles, 4D flow CMR measurements also showed a strong quadratic relationship (adjusted R^2^ = 0.86) between vorticity and energy dissipation rate (Fig. 4b). This was theoretically expected and the percentage of energy dissipation due to vorticity (21 ± 2%) agreed well with the idealized vortex ring. In the RV no differences were found for the energy dissipation mechanism between groups (Fig. 4c). In the LV the percentage of energy dissipation due to vorticity was less in the stenosis group (*p* = 0.02 vs sham, *p* = 0.04 vs intervention) indicating increased energy dissipation from surface deformation (Fig. 4c). The mechanism of LV energy dissipation in the intervention group was not different than the sham control group.

## Discussion

The results of this study reveal previously unknown effects of unilateral PAS on ventricular mechanics. In this animal model, 4D flow CMR detected abnormal and inefficient flow in both the RV and the LV associated with untreated PAS. Stent interventions to relieve PAS normalized both RV and LV KE, vorticity and energy dissipation. These results are clinically relevant as they demonstrate that in addition to decreasing RV afterload and improving PA blood flow distributions [10] successful PA stent interventions improve bi-ventricular flow dynamics that should contribute to the long-term myocardial health.

While alterations in cardiac flow patterns and parameters seem nearly ubiquitous in cardiovascular disease, few studies have shown the capability of CHD interventions to correct changes in intra cardiac flows. In a swine CHD model of pulmonary valve insufficiency and unilateral PAS Petit et al., demonstrated that relief of the PAS with stent interventions reduced pulmonary regurgitation and improved RV systolic function [30]. This frequently cited animal study is used to justify aggressive interventional management of PAS in patients with pulmonary valve insufficiency in hopes of slowing RV dilation and preserving ventricular function. Through application of more advanced 4D flow CMR techniques Sjoberg et al. [31] demonstrated a decrease in RV systolic and diastolic KE in adult Tetralogy of Fallot (TOF) patients following surgical pulmonary valve replacement that corresponds with a decrease in RV volumes (RV EDV, 164 ± 18–116 ± 18 ml/m^2^). This was the first CHD study to quantify KE during the entire cardiac cycle and following surgical pulmonary valve replacement KE was comparable to controls in both ventricles. While our left PAS model should have milder changes in RV mechanics than tetralogy of Fallot (TOF) patients with severe pulmonary regurgitation, the results of the current study similarly demonstrate that PA stent interventions normalized the ventricular flow measures of KE, vorticity and energy dissipation in the ventricles to levels similar to sham controls. This normalization in ventricular flow mechanics in combination with normal PA pressures documented at catheterization should reduce the energetic demands of the myocardium and we hypothesize this contributes to clinical improvements seen following successful PAS interventions [8–10]. Our previous swine PAS stenting study demonstrated that in response to dobutamine stress testing, the stent intervention animals increased cardiac output similar to sham controls while under the same stress conditions CO actually decreased in untreated PAS [20]. The proposed mechanisms responsible for clinical improvement in exercise capacity after relief of branch PAS include improved ventilation-perfusion matching, reduced RV afterload and improved stroke volume response to meet increased metabolic demands [8–10]. The current study would suggest that preserved ventricular flow mechanics play an additional role in clinical improvement following successful PAS interventions. Further study is warranted, including the effects of pulmonary regurgitation.

Studies of heart failure patients have found that exercise capacity is related to LV direct flow KE [32] and LV KE fluctuations [33]. In addition to exercise capacity, LV KE in heart failure patients is moderately associated with heart failure symptoms, serum markers of ventricular remodeling, and myocardial energetics [32]. While PA stent interventions improved ventricular flow dynamics, it is interesting that the intervention group remodeled to have higher EF compared to the sham and PAS controls. It appears that ventricular remodeling in the intervention group occurred to maintain normal ventricular flow and mechanical homeostasis for the myocardium. The effects of this remodeling seem beneficial (higher EF and improved flow efficiency) and consistent with improved exercise capacity in humans following relief of PAS.

4D flow CMR showed that the PAS group had abnormal and inefficient flow in the RV (Figs. 2 and 3) despite having similar heart rate, CI and EF values compared to sham controls. This finding was similar to 4D flow CMR results for TOF patients, who had RV KE inefficiency [34], elevated RV diastolic KE [31] and increased RV vortexes and vorticity [27, 35] compared to healthy subjects. Similarly, patients who have surgically repaired transposition of the great arteries have increased pulmonary artery vortices [36]. The changes in RV flow parameters are disease dependent. In swine pulmonary regurgitation increases RV KE and PAS decreases RV KE [37]. It is important to note that while Fernandes et al. found decreased RV KE in the pressure overloaded swine RV, the opposite of our finding, their PAS was surgically created at the level of the main PA and the resultant RV pressure overload was much greater [37].

We were surprised to find that LV flow was also altered in the PAS group when only the RV was directly affected by the stenosis and the degree of pressure elevation was mild. We would speculate These results seem similar to rTOF patients who despite having normal LV function exhibit decreased LV KE [31]. It has previously been shown that in pediatric PA hypertension patients, increased RV pressure decreases septal curvature which decreases LV torsion and ultimately LV function [38, 39]. We speculate that our results showing abnormal LV flow in the setting of mildly elevated RV afterload are early signs of the impact of septal mechanics on LV function. Again similar to our results following PAS stenting, a right side intervention, improved LV flow, pulmonary valve replacement in TOF patients improves LV KE compared to healthy subjects [31]. The detection of altered RV and LV flow parameters with only mild RV afterload and no changes in traditional functional strain parameters underscores the sensitivity of 4D flow CMR derived biomarkers to detect cardiac dysfunction.

Additional technical discussion of two points regarding ventricular flow analysis that are pertinent to this study (1. the mechanism of energy dissipation, 2. dimensional analysis) are included in Appendix 2.

### Limitations

Limitations of this work include the use of an animal model of CHD in place of human subjects as myocardial and PA response to catheter interventions may be species specific. The short time course may limit the degree of acquired disease. In addition, only a small number of subjects were studied per group and there was likely not sufficient power to detect differences between groups for metrics that had greater variability (e.g. RV energy dissipation and RV strain). Myocardial strain analysis only quantified circumferential strain and future studies should consider longitudinal strain which is a more reproducible measure. All measurements occurred under general anesthesia which is known to decrease myocardial contractility in swine. We cannot rule out the possibility that greater RVEF in the intervention group could be a result of differential response to anesthesia rather than a true difference in myocardial function. This study also used only male swine so we were unable to assess for any potentially important sex differences [40].

## Conclusion

In a swine model of PAS, stent interventions normalized RV and LV flow mechanics as measured by 4D flow CMR. Additionally, we identified significantly inefficient RV and LV flow associated with unilateral branch PAS, even in the setting of normal heart rate, EF and EDV index. The stent intervention group may have achieved normal ventricular flow by increased EF. These results provide evidence that PAS impacts ventricular function in CHD in subtle, yet likely important ways. Successful PA stent interventions improve ventricular flow efficiency and may promote long-term health of the ventricle. This study also highlights the sensitivity of 4D flow CMR biomarkers to detect ventricular dysfunction and treatment response in a pediatric setting.

## Data Availability

The datasets used and/or analysed during the current study are available from the corresponding author on reasonable request.

## Appendices

### Appendix 1

See Table 4

**Table 4:** P-values for all statistical comparisons

	Sham vs Left PAS	Intervention vs Sham	Intervention vs Left PAS
BW (kg)	0.99	0.83	0.72
HR (BPM)	0.89	0.74	0.41
RV/(LV + S) (g/g)	0.67	0.94	0.38
RA pressure (mmHg)	0.08	0.64	0.007
RV systolic pressure (mmHg)	0.003	1.00	0.001
Mean MPA pressure (mmHg)	0.015	0.67	0.001
MPA pressure (sys/dia, mmHg)	0.002	0.47	0.000
RPA pressure (sys/dia, mmHg)	0.16	1.00	0.007
Distal LPA pressure (sys/dia, mmHg)	0.002	0.17	0.02
Proximal LPA pressure gradient (mmHg)	0.000	0.41	0.000
PCWP (mmHg)	0.64	0.21	0.03
Proximal LPA:Ao diameter (mm/mm)	0.000	0.03	0.000
LPA 1: Ao diameter (mm/mm)	0.000	0.05	0.004
LPA 2: Ao diameter (mm/mm)	0.006	0.90	0.003
L lung perfusion (%)	0.000	0.07	0.000
CI (L/min/m^2^)	0.72	0.08	0.37
RV SV/BSA (mL/m^2^)	0.42	0.48	0.04
RV ESV/BSA (mL/m^2^)	0.38	0.11	0.005
RV EDV/BSA (mL/m^2^)	0.84	0.72	0.99
RV EF (%)	1.00	0.06	0.03
LV SV/BSA (mL/m^2^)	0.54	0.06	0.004
LV ESV/BSA (mL/m^2^)	0.86	0.009	0.002
LV EDV/BSA (mL/m^2^)	0.63	0.87	0.81
LV EF (%)	0.89	0.06	0.16
LV Peak strain (%)	–	–	–
LV Peak diastolic strain rate (%/s)	–	–	–
LV peak systolic strain rate (%/s)	–	–	–
RV peak strain (%)	–	–	–
RV peak diastolic strain rate (%/s)	–	–	–
RV peak systolic strain rate (%/s)	–	–	–
Sys. RV KE/SV	0.07	0.93	0.06
Dia. RV KE/SV	–	–	–
RV vorticity	–	–	–
RV dissipation/SV	0.34	0.96	0.15
Sys. LV KE/SV	0.008	0.85	0.007
Dia. LV KE/SV	0.02	0.67	0.03
RV vorticity	0.008	0.65	0.01
RV dissipation/SV	0.08	1.00	0.01
Sys. RV KE (n.d.)	0.98	0.59	0.04
Dia. RV KE (n.d.)	0.15	0.54	0.01
RV vorticity (n.d.)	0.12	0.33	0.004
RV dissipation (n.d.)	–	–	–
Sys. LV KE (n.d.)	0.01	0.31	0.000
Dia. LV KE (n.d.)	0.24	1.00	0.04
LV vorticity (n.d.)	0.01	0.03	0.000
LV dissipation (n.d.)	0.37	0.46	0.004
RV % Dissipation from vorticity	–	–	–
LV % Dissipation from vorticity	0.02	0.63	0.04

### Appendix 2

In Appendix 2 we further expand our discussion to include two important technical points about ventricular flow analysis: 1. the mechanism of energy dissipation and 2. dimensional analysis. First, we calculated the percentage of energy dissipation directly due to vorticity as a novel metric of ventricular flow-structure interactions. Statistical correlations between flow parameters and ventricular kinematics [40] are useful but may only be associations and lack a direct physical basis. We propose a new metric by recognizing that viscous energy dissipation is due to two components, vorticity and surface strain rate [41]. The surface-strain rate component of energy dissipation is zero for flows with stationary walls and for these flows energy dissipation is solely due to vorticity. The percentage of energy dissipation directly due to vorticity is then a metric that will decrease if structural motion is excessively causing energy losses. We found percentage of energy dissipation from vorticity did not change in the right ventricle (RV) but in the left ventricle (LV) the pulmonary artery stenosis (PAS) group had a lower percentage of energy dissipation from vorticity. These results suggest that in PAS, the LV wall and mitral valve motion are excessively contributing to energy dissipation compared to the sham and intervention groups. The percentage of energy dissipation from vorticity could be a biomarker of the impact of ventricular wall and atrioventricular valve kinematics on ventricular flow efficiency in other diseases as well.

Second, we used dimensional analysis to calculate dimensionless ventricular flow parameters. It is important to normalize cardiovascular measurements given anatomical and functional differences between subjects, however 4D flow CMR derived metrics such as kinetic energy and vorticity are not systematically normalized. As ventricular flow analysis sits at a unique intersection between physiology and fluid dynamics, careful consideration must be given to the proper normalization of parameters. In this study calculating dimensionless parameters to account for subject variability in stroke volume, HR and ventricular volume improved the sensitivity of ventricular flow analysis to detect statistical differences in flow patterns between groups. Heart rate, stroke volume and ventricular volume can change in a variety of cardiovascular diseases and calculating dimensionless parameters may aid ventricular flow analysis in other diseases as well.

## Abbreviations

BSA: Body surface area
bSSFP: Balanced steady state free precession
CHD: Congenital heart disease
CI: Cardiac index
CMR: Cardiovascular magnetic resonance
CO: Cardiac output
CT: Computed tomography
EDV: End-diastolic volume
EF: Ejection fraction
ESV: End-systolic volume
IVS: Interventricular septum
KE: Kinetic energy
LPA: Left pulmonary artery
LV: Left ventricle/left ventricular
LVEF: Left ventricular ejection fraction
PA: Pulmonary artery
PAS: Pulmonary artery stenosis
PCWP: Pulmonary capillary wedge pressure
RHC: Right heart catheterization
RPA: Right pulmonary artery
RV: Right ventricle/right ventricular
RVEF: Right ventricular ejection fraction
SV: Stroke volume
TOF: Tetralogy of Fallot
